# Modified Delphi procedure-based expert consensus on endpoints for an international disease registry for Metachromatic Leukodystrophy: The European Metachromatic Leukodystrophy initiative (MLDi)

**DOI:** 10.1186/s13023-022-02189-w

**Published:** 2022-02-14

**Authors:** Daphne H. Schoenmakers, Shanice Beerepoot, Sibren van den Berg, Laura Adang, Annette Bley, Jaap-Jan Boelens, Francesca Fumagalli, Wim G. Goettsch, Sabine Grønborg, Samuel Groeschel, Peter M. van Hasselt, Carla E. M. Hollak, Caroline Lindemans, Fanny Mochel, Peter G. M. Mol, Caroline Sevin, Ayelet Zerem, Ludger Schöls, Nicole I. Wolf

**Affiliations:** 1 Amsterdam Leukodystrophy Center, Department of Child Neurology, Emma Children’s Hospital, Amsterdam University Medical Centers, Vrije Universiteit Amsterdam, Amsterdam Neuroscience, Amsterdam, The Netherlands; 2 Department of Endocrinology and Metabolism, Amsterdam University Medical Centers, Amsterdam, The Netherlands; 3 Medicine for Society, Platform at Amsterdam University Medical Centers, Amsterdam, The Netherlands; 4 Center for Translational Immunology, University Medical Center Utrecht, Utrecht, The Netherlands; 5 Nierkens and Lindemans group, Princess Máxima Center for pediatric oncology, Utrecht, The Netherlands; 6 Division of Neurology, Children’s Hospital of Philadelphia, Philadelphia, Pennsylvania USA; 7 University Children’s Hospital, University Medical Center Hamburg Eppendorf, Hamburg, Germany; 8 Stem Cell Transplantation and Cellular Therapies Program, Department of Pediatrics, Memorial Sloan Kettering Cancer Center, New York, NY 10065 USA; 9 San Raffaele Telethon Institute for Gene Therapy (SR-Tiget); IRCCS, San Raffaele Scientific Institute, Milan, Italy; 10 Zorginstituut Nederland (Dutch Health Care Institute), Diemen, The Netherlands; 11 Division of Pharmacoepidemiology and Clinical Pharmacology, Utrecht University, Utrecht, The Netherlands; 12 Centre for Inherited Metabolic Diseases, Copenhagen University Hospital (Rigshospitalet), Copenhagen, Denmark; 13 Department of Paediatric Neurology and Developmental Medicine, University Children’s Hospital, Tübingen, Germany; 14 Department of Pediatric Metabolic Diseases, Wilhelmina Children’s Hospital, University Medical Center Utrecht, Utrecht University, Utrecht, The Netherlands; 15 Department of Pediatrics, Wilhelmina Children’s Hospital, University Medical Center Utrecht, Utrecht University, Utrecht, The Netherlands; 16 INSERM U 1127, CNRS UMR 7225, Sorbonne Universités, UPMC Univ Paris 06 UMR S 1127, Institut du Cerveau Et de La Moelle Épinière, ICM, 75013 Paris, France; 17 Department of Genetics, Center for Neurometabolic Diseases, AP-HP, La Pitié-Salpêtrière University Hospital, 47 Boulevard de l’Hôpital, 75013 Paris, France; 18 Department of Clinical Pharmacy and Pharmacology, University Medical Center Groningen, University of Groningen, Groningen, The Netherlands; 19 Dutch Medicines Evaluation Board, Utrecht, The Netherlands; 20 NeuroGenCell, Institut du Cerveau et de la Moelle Épinière, ICM, Inserm U 1127, CNRS UMR 7225, Sorbonne Université, Paris, France; 21 Bicêtre Hospital, Neuropediatrics Unit, Le Kremlin Bicêtre, Paris, France; 22 Pediatric Neurology Institute, Tel-Aviv Sourasky Medical Center, Tel-Aviv, Israel; 23 Sackler Faculty of Medicine, Tel Aviv University, Tel-Aviv, Israel; 24 Department of Neurology and Hertie-Institute for Clinical Brain Research, University of Tübingen, 72076 Tübingen, Germany; 25 German Center of Neurodegenerative Diseases, 72076 Tübingen, Germany

**Keywords:** Rare disease registry, Rare diseases, Metachromatic leukodystrophy, MLD, Delphi procedure

## Abstract

**Background:**

Metachromatic Leukodystrophy (MLD) is a rare lysosomal disorder. Patients suffer from relentless neurological deterioration leading to premature death. Recently, new treatment modalities, including gene therapy and enzyme replacement therapy, have been developed. Those advances increase the need for high-quality research infrastructure to adequately compare treatments, execute post-marketing surveillance, and perform health technology assessments (HTA). To facilitate this, a group of MLD experts started the MLD initiative (MLDi) and initiated an academia-led European MLD registry: the MLDi. An expert-based consensus procedure, namely a modified Delphi procedure, was used to determine the data elements required to answer academic, regulatory, and HTA research questions.

**Results:**

Three distinct sets of data elements were defined by the 13-member expert panel. The minimal set (n = 13) contained demographics and basic disease characteristics. The core set (n = 55) included functional status scores in terms of motor, manual, speech and eating abilities, and causal and supportive treatment characteristics. Health-related quality of life scores were included that were also deemed necessary for HTA. The optional set (n = 31) contained additional clinical aspects, such as findings at neurological examination, detailed motor function, presence of peripheral neuropathy, gall bladder involvement and micturition.

**Conclusion:**

Using a modified Delphi procedure with physicians from the main expert centers, consensus was reached on a core set of data that can be collected retrospectively and prospectively. With this consensus-based approach, an important step towards harmonization was made. This unique dataset will support knowledge about the disease and facilitate regulatory requirements related to the launch of new treatments.

**Supplementary Information:**

The online version contains supplementary material available at 10.1186/s13023-022-02189-w.

## Introduction

**Fig. 1:**
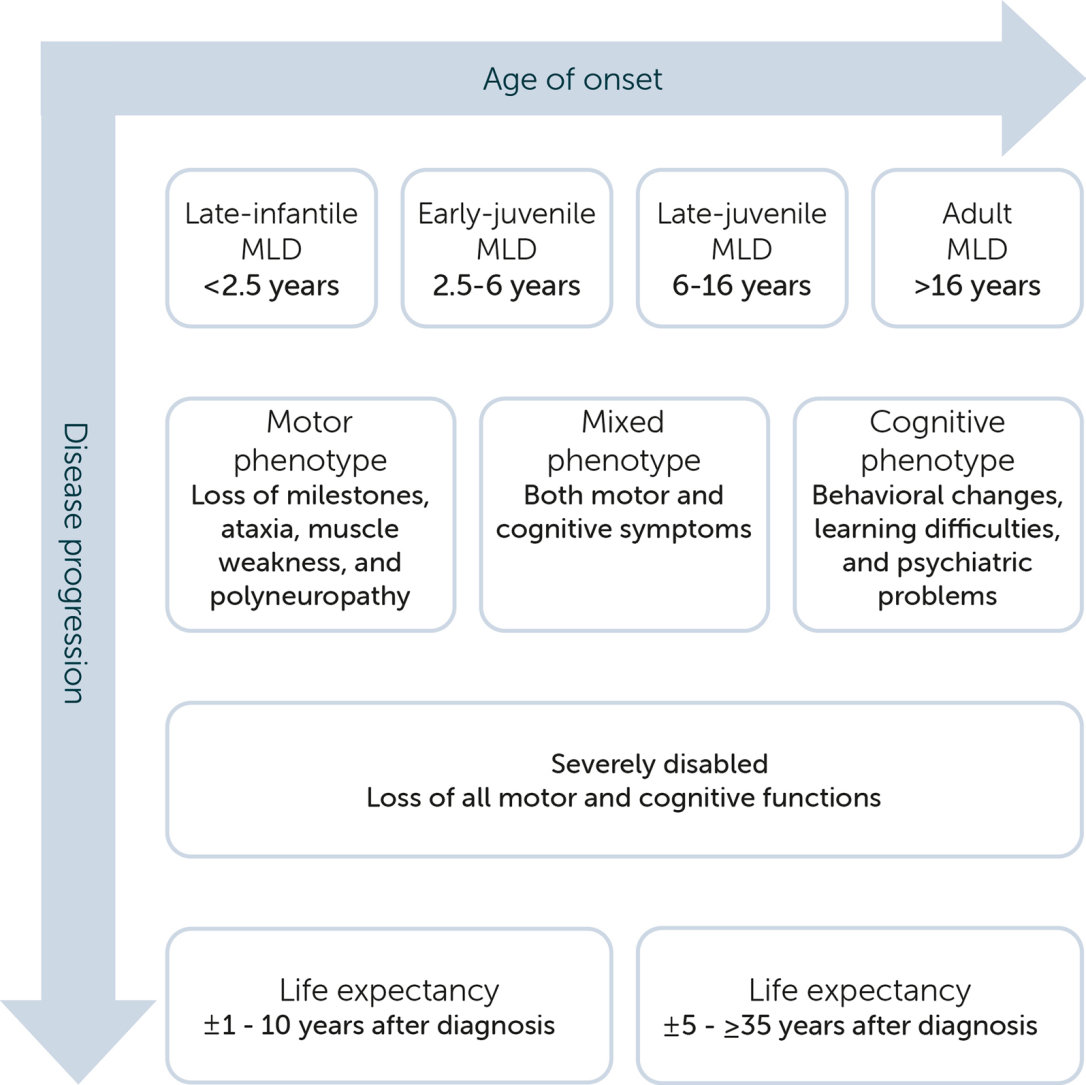
Clinical spectrum of MLD

Metachromatic leukodystrophy (MLD, OMIM 250,100 and 249,900) is an autosomal recessively inherited lysosomal storage disorder with an estimated birth prevalence of 1 in 40.000 [1]. The disease is caused by pathogenic variants in the *ARSA* gene, encoding the lysosomal enzyme arylsulfatase A (ASA), or, more rarely, by variants in the *PSAP* gene, encoding the activator protein saposin B [2, 3]. The deficiency of either one of the two results in sulfatide accumulation in multiple organs, including central and peripheral nervous system, gall bladder, kidneys, and liver. Myelin sheaths of the central and peripheral nervous system are especially affected, resulting in progressive demyelination. This causes neurological deterioration and, if untreated, eventually leads to death [4]. Based on the age of symptom onset, four clinical MLD phenotypes are distinguished: late-infantile (< 2.5 years), early-juvenile (2.5–6 years), late-juvenile (6–16 years), and adult (> 16 years) MLD [5]. Symptom-onset at a younger age is generally associated with a faster disease progression and shorter life expectancy, as shown in Fig. 1 [2, 5, 6].

Supportive care, including treatment of spasticity, tube feeding, and psychological support is important for all symptomatic patients with MLD. MLD cannot be cured. Causal treatment targeting the enzyme deficiency is an option for a subset of patients. In presymptomatic or early disease stages, patients are eligible to receive causal treatment, including allogeneic hematopoietic stem cell transplantation (HSCT), which provides a clinical and survival benefit for patients with early-juvenile, late-juvenile and adult MLD. Causal treatment outcomes vary. In some patients disease progression stagnates or slows down, in others, treatment is not effective, and symptoms get worse [7–10]. Over the last decade, new treatments for MLD have emerged. Recently, autologous HSC-based gene therapy (GT) has been authorized in the European Union for pre-symptomatic patients with late-infantile and pre- and early-symptomatic patients with early-juvenile MLD [11–13]. Other new therapeutic options, such as intrathecal enzyme replacement therapy, are being investigated in clinical trials [14]. Importantly, treatment eligibility strongly depends on phenotype and disease stage as visualized in Table 1.

**Table 1:** Current therapeutic options

	Late-infantile			Early-juvenile			Late-juvenile			Adult		
Disease stage	Pre-	Early	Late	Pre-	Early	Late	Pre-	Early	Late	Pre-	Early	Late
Supportive care	✔	✔	✔	✔	✔	✔	✔	✔	✔	✔	✔	✔
HSCT	?	✖	✖	✔	✔	✖	✔	✔	✖	✔	✔	✖
Ex vivo GT	✔	✖	✖	✔	✔	✖	Trial	Trial	✖	?	?	✖
ERT	?	Trial	Trial	?	?	?	?	?	?	?	?	?

*Trial* Currently investigated

✔ Eligible

✖ Not eligible

? Not investigated or debatable indication

New treatments create hope for patients and families, but also harbor scientific and regulatory hurdles. Due to the rarity of MLD it remains challenging to perform registrational trials. Those trials require considerable sample sizes and uniformly collected clinical data of both treated and untreated patients. In addition, long-term follow-up is often indispensable to show a lasting effect on clinically relevant endpoints [4, 10, 12]. Indeed, the recently authorized GT Libmeldy (Orchard Therapeutics BV) is subject to additional monitoring and the marketing authorization holder has the obligation to prospectively characterize long-term efficacy and safety through a registry [15]. Another obstacle on national level is that in most countries such new and expensive therapies are scrutinized for relative and/or cost-effectiveness before a decision on reimbursement will be made. Because of differences in these national processes this may lead to unequal access between EU countries.

To overcome those challenges, international and uniform data collection is needed. An academic-led international disease registry could provide the required infrastructure for this purpose. To ensure its success, the registry should ideally be based on patient-centered multi-stakeholder collaborations [16–18]. Along these lines, drug regulators, HTA bodies/payers, drug developers, and academia can join forces to improve the process of rare disease research, drug development, and post-marketing studies [19, 20]. From this point of view, the MLD initiative (MLDi) which is initiated by a group of MLD experts from international leukodystrophy centers, launched an academia-led European disease registry for MLD. In this registry, all participating centers are data controllers, while the Amsterdam UMC also acts as processor, according to the GDPR. A crucial step in establishing a registry is deciding which data elements should be collected. For this reason, a consensus procedure with an international multidisciplinary expert panel was organized. In this paper, we provide an overview of the modified Delphi procedure used for this goal and the resulting list of data elements.

## Methods

**Fig. 2:**
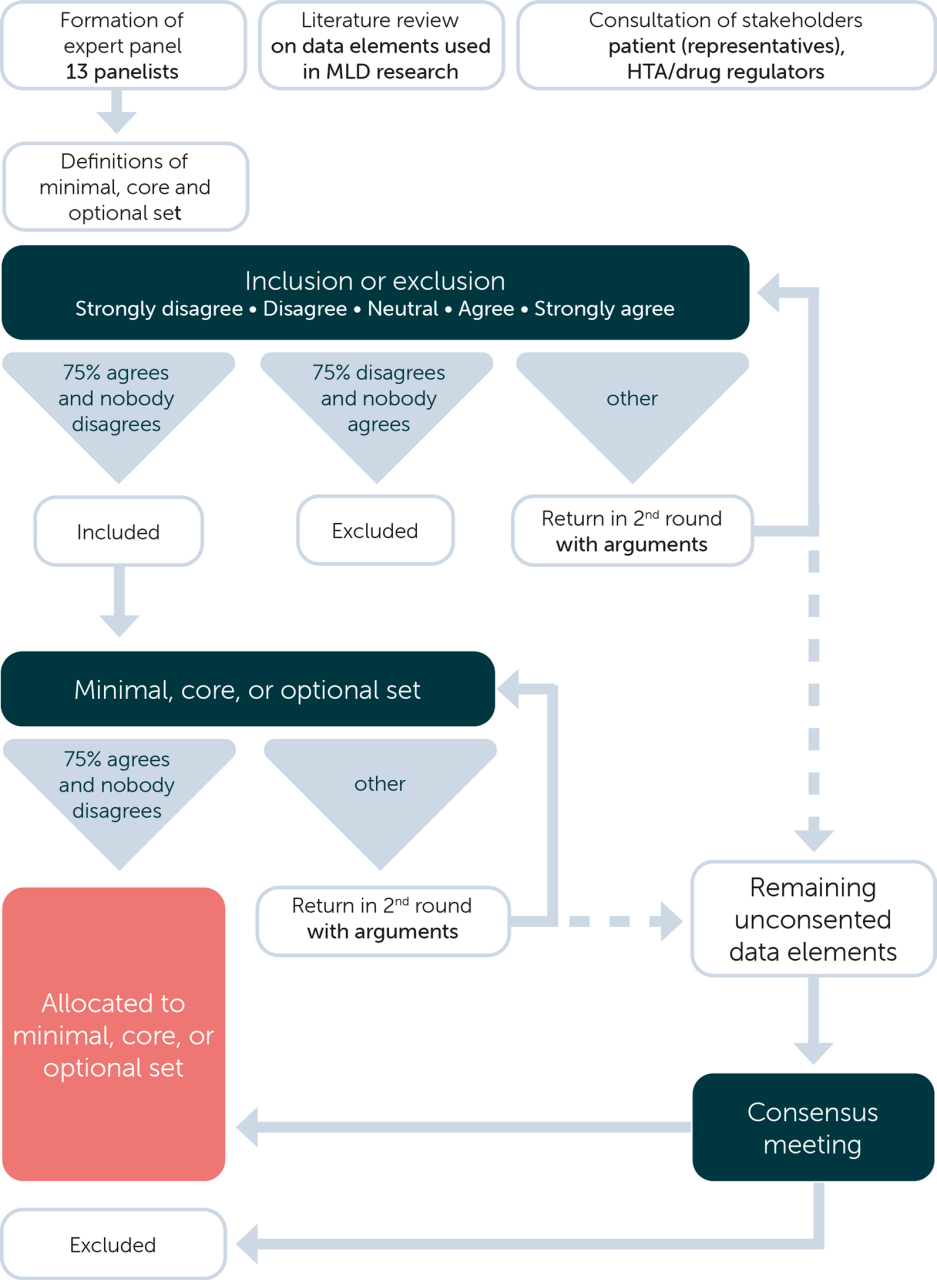
Methodological overview of the modified Delphi study

To achieve consensus on which registry data elements should be included, a modified Delphi procedure was used. Input for this procedure was provided by consulted stakeholders, expert panelists and a literature review. The original Delphi study uses multiple rounds of questionnaires aimed at reaching a consensus on a certain subject. The ‘modified’ approach starts with a structured questionnaire based upon a review of current literature and clinical trial databases, reducing the number of rounds needed [21, 22]. The systematic literature review, together with the view of the consulted stakeholders, was sent to the expert panel before the start of the procedure. A schematic overview of the method is provided in Fig. 2.

### Consultation of stakeholders

Patient and caregiver input was invited by a questionnaire that was digitally sent to patients and caregivers before the Delphi procedure started. The questionnaire consisted of four open questions exploring their view on essential data elements for the MLDi registry. One patient with adult MLD and three families, all recruited in the Amsterdam Leukodystrophy Center (ALC), were invited to participate and responded to the questionnaire. The answers were qualitatively analyzed and discussed in the Delphi procedure.

Representatives from the Dutch Healthcare Advisory Institute (Zorginstituut Nederland) were consulted to evaluate the suitability of the set of data elements for its application in health technology assessments (HTA). In addition, regulators from the Dutch Medicines Evaluation Board were consulted. All perspectives served as input in the procedure.

### Expert panel

Members of the European Reference Network on Rare Neurological Diseases (ERN-RND) guideline group on MLD (n = 10) were invited to participate in the expert panel. Additionally, physicians with expertise on MLD care and research (n = 9) were invited. This was defined as working in a dedicated leukodystrophy center in Europe including Israel, and taking part in clinical trials on MLD. Our focus is Europe, to start the registry in a relatively uniform legal and geographical region. In addition, an in New York employed HSCT expert from Utrecht, The Netherlands, and an MLD natural history expert from Philadelphia, USA were invited. Five invited physicians did not respond to the invitation, and one was not able to participate in the questionnaires and meetings. The final panel consisted of 13 experts from 11 different centers worldwide (Denmark, France, Germany, Israel, Italy, Netherlands, and United States of America), representing child neurologists (n = 6), neurologist (n = 3), pediatricians (n = 2), and transplant specialists (n = 2).

### Literature and database review

A systematic literature search of the PubMed, Cochrane Library, and Embase databases was performed to identify relevant publications reporting potential MLD data elements between 2000 and May 2020. The search strings are reported in Additional file 1. Titles and abstracts were screened for predefined criteria by two physician reviewers (DS, SB). English-written, peer-reviewed studies in human subjects were included for full-text analysis. Disagreements on including or excluding a study were resolved by consensus-based discussion. Full texts were reviewed for eligibility and all reported data elements were collected. In addition to this literature search, we searched clinicaltrials.gov and clinicaltrialsregister.eu for recent clinical trials. Cross-referencing was performed to identify extra studies focusing on clinical endpoints. The collected data elements were organized and clustered into nine categories.

The literature search identified 472 studies of which 357 remained after removing duplicates. Title and abstract screening led to exclusion of 297 studies (Additional file 2). The full-text evaluation led to the exclusion of eight additional studies. Eleven relevant studies were identified through cross-referencing. This resulted in a total of 67 eligible studies (39 retrospective studies, 7 clinical trials, 5 prospective studies, 5 qualitative studies, 4 trial protocols, 4 reviews, 2 validation studies, and 1 case report) to collect relevant study variables. An overview of the included studies is provided in Additional file 3: Tables S2 and S3.

The study variables used, including patient characteristics, diagnostic tests, and clinical outcomes, were extracted from the 67 studies. The website of the European Rare Disease Platform (EU-RD platform) of the European Commission (EC) was consulted. In addition, the common set of data elements defined by the European Reference Network on Rare Neurological Disorders (ERN-RND) was added. In total, 178 different variables were identified. After removing duplicates and critical evaluation of the relevance by three physician reviewers (DS, SB, NW) this number was reduced to 123 variables.

### Definition of distinct sets of data elements

Based on the (draft) guideline on registry-based studies [23, 24], the information provided by the EU-RD Platform [25], and the experiences of panelists we decided to define three distinct sets of data elements:

1. Minimal data elements: mandatory to collect for every included patient. This means that a patient cannot be included in the MLDi registry if data on one or more of these elements are missing.
2. Core data elements: essential for the purpose of the registry. Those data elements are strongly encouraged but not mandatory to collect. This set aims to uniformly collect patient characteristics that were considered particularly important with respect to natural history and treatment research.
3. Optional data elements: considered of interest to a subset of patients or useful for some stakeholders. Those data elements are of additional value but are deemed less important or generate more heterogeneous data compared to the core data elements. Moreover, standardization is desired for some of these data elements before they become core data elements.

The distinct sets of data elements were technically implemented using a gradient for importance. For the minimal data elements, a ‘requiring to complete’ validation was used. Capturing data started with the minimal data elements, and was followed by core and optional data elements.

### Modified Delphi Study

The questionnaires were presented as online surveys using SurveyMonkey (SurveyMonkey Inc., 2021). Panelists were asked to indicate on a 5-point Likert scale whether they agreed to include a data element in the registry. Providing argumentation for a decision was encouraged, as well as suggesting additional data elements. In addition, panelists had to classify the items as core or optional data elements. After the first round, the responses were analyzed. Consensus was reached when at least 75% of the panelists agreed on the inclusion of that data element, and no one disagreed. A data element was removed when at least 75% of the panelists disagreed on the inclusion or less than 25% agreed on the inclusion of that data element. Data elements on which no consensus was reached returned in a second survey, containing the anonymized scores and comments of the first survey. The remaining unconsented data elements were subject to a plenary discussion during the two-part online consensus meeting. The video conferencing platform Zoom (Zoom Video Communications, Inc., 2021; version 5.6.7) and the real-time polling software Slido (Cisco Systems, Inc, 2021, version 38.76.1) were used for the meeting.

## Results

### Data elements

**Fig. 3:**
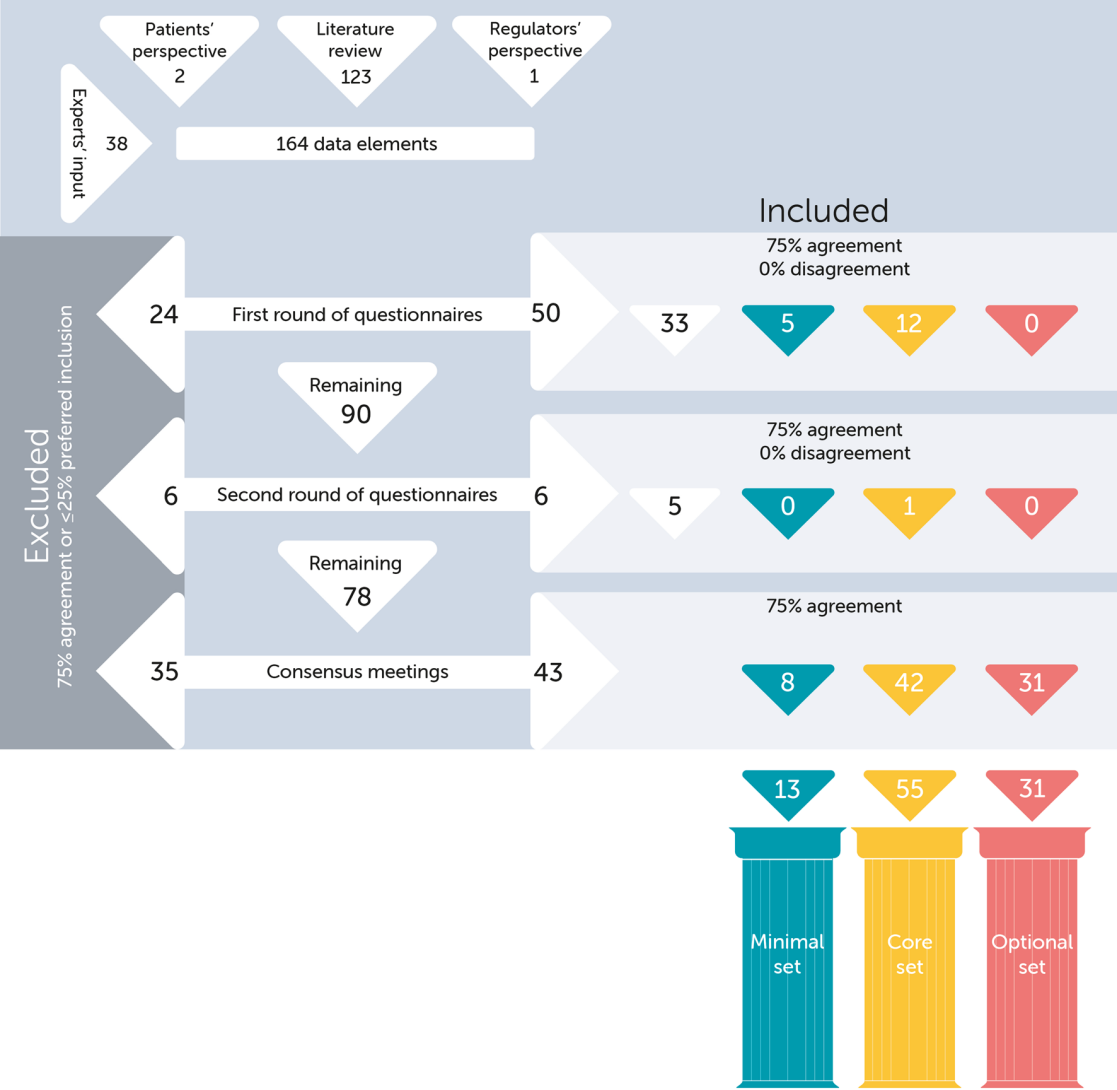
Flow chart of data elements in the modified Delphi study. 164 data elements were discussed leading to inclusion of 99 after two rounds of questionnaires and two consensus meetings. Two decisions are visualized, (1) inclusion/exclusion and (2) minimal/core/optional. White corresponds to unconsented or excluded data elements, or data elements that are included but still need to be allocated to the minimal/core/optional set. Blue corresponds to the minimal set. Yellow corresponds to the core set. Coral red corresponds to the optional set

A total of 164 data elements, of which 123 were extracted from literature and the remaining 41 suggested by patient representatives, regulators, and the expert panel, were reviewed by the expert panel. Eventually, 13 minimal data elements were defined, 55 core data elements, and 31 optional data elements (Fig. 3). The complete sets are added in Table 2 and Additional file 4: Tables S4 and S5. Below, we discuss the most important and remarkable outcomes of the procedure.

**Table 2:** Minimal set

Minimal data element	Coding
Approximate date of birth	mm/yyyy
Sex at birth	Male, female, unknown
Survival status	Alive, deceased, loss to follow-up, opted-out
	> date of death/loss to follow-up/opted-out
Name or country of specialized center	Specify center
Confirmed diagnosis (checkboxes)	Yes > genetically + clinically, enzymatically + genetically, enzymatically + urinary sulfatides
OMIM diagnosis	250,100, 249,900, 272,200
Approximate date of diagnosis (age at diagnosis)	mm/yyyy if unknown: antenatal, at birth, childhood, adult
Approximate date at symptom manifestation (age at symptom onset)	Pre-symptomatic, age in years and months
Relevant other diagnosis/comorbidity	No, Yes > specify other (inherited) important conditions/prenatal history
Inclusion of the patient in the registry is allowed	Yes consent was given, no but exceptional* circumstances apply
Agreement to be contacted for research purposes	Yes, no, missing, not applicable
Biological sample	Yes, no, unknown
Link or information to a biobank	If applicable: free text

*Exceptional circumstances include consent was given for another registry/database/reuse of data, or a patient is deceased and inclusion in the registry will likely not harm the patient or his/her relatives

### Minimal elements

The minimal set (Table 2) is obligatory to collect to make sure a unique patient is included and to characterize important subgroups of patients. Most elements of the set of common data elements published by the EU-RD Platform were included [25]. Only the ‘International Classification of Functioning and Disability’ was omitted because other functional systems were preferred and the ‘undiagnosed case’ data element is not relevant in this disease registry. Several key indicative demographics, disease characteristics, and consent information are part of this minimal set, for example an approximate of the date of birth (month/year), sex at birth, survival status, age at diagnosis, age at onset, and relevant comorbidities. In addition, three phenotype numbers according to the Online Mendelian Inheritance in Man (OMIM) will be collected, including the numbers for both genes associated with MLD (250100, 249900) and the phenotype number for multiple sulfatase deficiency (MSD, 272200). As long as no separate registry is available for MSD, it was decided to collect those patients in the MLDi registry for the time being.

### Genetics

The causal genetic variants (either in *ARSA* or in *PSAP*) were defined as part of the core data set, as the expert panel unanimously agreed on the need for more research into genotype–phenotype correlations. Common variants, based on the publications of Cesani et al. 2016 and Beerepoot et al. 2020, can be selected from a predefined list in the registry (Additional file 4). [2, 3] Other variants can be provided in a free text field, preferably on DNA level, e.g. 'c.256C > T' notation.

### Brain MRI

MRI of the brain plays a pivotal role for both diagnosis and treatment decisions in MLD. The expert panel consented to inclusion of the total MLD-Loes score as a core data element [26]. The total adapted MLD-Loes score was added as an optional data element [12]. The expert panel aims to store full MRIs in the registry and is looking into technical possibilities regarding anonymizing, storing, and displaying of MRIs.

### Clinical scores

The expert panel agreed that the cornerstone of disease monitoring and registering treatment response is a set of clinical scores, to summarize a patient’s functional state in terms of motor, speech, eating, and manual abilities. Included were those clinical scores that are comparably easy to collect and regularly used in MLD, including the Gross Motor Function Classification for MLD (GMFC-MLD) and Expressive Language Function Classification for MLD (ELFC-MLD), both validated for MLD [27, 28]. Other included scores were the Eating and Drinking Ability Classification System (EDACS) [29] and Manual Ability Classification System (MACS) [30], which were originally developed for patients with cerebral palsy (CP). As MLD, although being a progressive disorder, does share some of the impairments with CP and there are no comparable scales validated in leukodystrophy patients, we assumed that the use of EDACS and MACS is justified in MLD. The clinical scores are summarized in Table 3.

**Table 3:** Clinical scores and measurement tools recommended to collect in MLD patients

Clinical scoring systems	Versions	Age groups (years)	Population (development and validation)	Used in MLD before
GMFC-MLD [27]	1 version	1.5–18, [>18]	MLD patients	Yes [7, 9–12, 31–40]
ELFC-MLD [28]	1 version	1.5–18, [>18]	MLD patients	Yes [10, 27, 28, 34]
EDACS [29]	1 version	2–21, [>21]	CP patients	Yes [40–42]
MACS [30]	MACS Mini-MACS	4–18, [>18] 1–4	CP patients	Yes, unpublished

Measurement tools	Versions	Age groups (years)	Population (development and validation)	Used in MLD before
IQ	Many different tools	> 2.5	Non-specific populations	Yes, frequently*
*MMSE* [43]	1 version	> 18	Non-specific adult populations	Yes, unpublished
*GMFM-88* [44]	GMFM-88 & GMFM-66	0.4–16, [> 16]	Non-specific pediatric populations	Yes [45–47]
*SARA* [48]	1 version	[≥ 3], > 8	Different SCA- and non-SCA populations with ataxia	Yes, unpublished

PROMs	Versions	Age groups (years)	Population (development and validation)	Used in MLD before
EQ5D/5L and EQ5D-Y [49–51]	Modes of administration (self-assessment, interviewer-administered, and proxy), age groups	4–7, 8–15, > 15	Non-specific populations	No
*HUI3* [52]	Modes of administration (self-assessment, interviewer- administered, and proxy)	> 1	Non-specific populations	No
*PedsQL* [53, 54]	Age groups	2–4, 5–7, 8–12, 13–18	Non-specific pediatric populations	Yes [55]**

[between square brackets] = not validated. Underlined = core data element, italic = optional data element

*No references added because a lot of heterogeneity in used scales and sometimes only total IQ was reported without the used scale

**Only the PedsQL Family Impact Module is used

*CP* cerebral palsy, *EDACS* Eating- and Drinking Ability Classification System, *ELFC-MLD* Expressive Language Function Classification for MLD, *EQ5D/5L* EuroQoL5D/5L, *EQ5D-Y* EuroQoL5D-Youth, *GMFC-MLD* Gross Motor Function Classification for MLD, *GMFM-88* Gross Motor Function Measure-88, *HUI3* Health Utilities Index 3, *IQ* intelligence quotient, *MACS* Manual Ability Classification System, *MLD* metachromatic leukodystrophy, *MMSE* Minimal Mental State Examination, *PedsQL* Pediatric Quality of Life Inventory, *PROMs* Patient Reported Outcome Measures, *SARA* Scale for the Assessment and Rating of Ataxia, *SCA* spinocerebellar ataxia

### Other instruments

There was consensus to collect the intelligence quotient (IQ) as a core data element. No strong opinion on the IQ scale used was expressed. Both the total IQ score and the IQ subscores were considered important and will be collected as core data elements. The Gross-Motor Function Measure-88 (GMFM-88) [44] and the Scale for the Assessment and Rating of Ataxia (SARA) [48] were added to the optional set of data elements. The GMFM-88 can be used to comprehensively assess gross motor function and is also yet used as outcome in clinical trials. The expert panel concluded that the SARA is not widely used in MLD. Nevertheless, ataxia is frequent in MLD, and SARA is a validated scale to semi-quantify this sign. So, it was placed in the optional set.

### Patient-/proxy reported outcome measures

Patient-reported outcome measures (PROMs) were partly included in the core set (Table 3) and partly in the optional set (Additional file 4: Table S5). The patient-reported dataset (PRD) consists of four parts.

#### Quality of life and functioning in daily life

The importance of collecting a widely used quality of life (QoL) scale was stressed by HTA authorities. The expert panel concluded that none of the QoL measurement tools is commonly used within the leukodystrophy field. Nevertheless, the expert panel agreed with the HTA authorities to include at least one QoL scale. The expert panel achieved consensus on the collection of the EuroqolQ5D (EQ5D/5L; EQ5D-Y) as a core data element. This is a widely used QoL assessment tool and is preferred by HTA authorities. In addition, the Health Utilities Index (HUI) and the Pediatric Quality of Life Inventory (PedsQL) were added to the optional data elements, because these scales have been used in leukodystrophies before [55–57]. No consensus was reached on the collection of a classic activities of daily living (ADL) scale for adults, such as the Barthel index or the iADL. Currently, none of the ADL scales are widely used in MLD. As the QoL scales selected for the registry, such as EQ5D/5L, do contain some aspects of ADL functioning, the panel decided not to include a dedicated ADL scale for the time being, but agreed that new insights or regulatory necessities may require the addition of an ADL scale in the future.

#### Irritability and happiness

The expert panel decided to include irritability and happiness, as suggested by the consulted patient representatives. Patient or proxies are asked to indicate the patient’s irritability and happiness by choosing between always, mostly, rarely, and never (Additional file 4: Tables S4 and S5). Irritability was considered to be easier assessable compared to a general state of happiness, therefore those two items were included in the core and optional set respectively.

#### Developmental milestones

The expert panel advised collecting information about the initial development of children, thus in late-infantile and juvenile patients. Developmental motor milestones, according to the World Health Organization (WHO) Multicentre Growth Reference Study will be registered in the core set, as patient-reported or clinical-reported data element [58] (Additional file 4: Table S4).

#### School career

To gain insight into the educational development of the patient, the expert panel decided to collect information on the school career as a patient-centered surrogate outcome. It was not deemed suitable as a core data element, because educational systems differ too much. It was added to the optional set as patient-reported data element (Additional file 4: Table S5).

### Treatment-related data elements

In causally treated patients, a wide range of data elements regarding treatment were consented to be collected. Technical treatment characteristics, among which type of conditioning regimen, donor type, and graft source, as well as treatment outcomes, such as enzyme activity after treatment and chimerism, were included. Adverse events related to the use of specific medicines should be collected. Since pharmaceutical companies usually have a strong pharmacovigilance department and will have an obligation to report on safety issues, a structure needs to be discussed with individual companies on how to arrange collection and (expedited) official reporting and evaluation of adverse events and other safety issues. These elements are presented in Additional file 4: Table S4 and will be collected as core data element for all treated patients.

### Peripheral neuropathy

The expert panel extensively discussed the collection of neurophysiological parameters regarding peripheral neuropathy. During the discussions, it became clear that the heterogeneity in the investigation methods and data are a significant hurdle for comparing crude nerve conduction measurements across centers. The panel therefore decided to collect ‘signs of polyneuropathy (yes/no)’ followed by the question ‘demyelinating polyneuropathy confirmed with EMG (yes/no)’ as an optional data element.

## Discussion

The importance of rare disease registries and their application in academic and regulatory research has been frequently stressed in literature. Registries can function as independent infrastructures that foster rare disease research, orphan drug development, and support regulatory decision-making [16, 19, 20, 59, 60]. A framework for rare disease registries has been suggested, containing recommendations on requirements for software technology, principles for data management, and governance structures, but overarching data elements on disease-specific outcomes have not been sufficiently defined [17, 23, 61–65]. In addition, EUnetHTA developed a tool, REQuesT, to assess registries’ methodological and organizational quality. This tool can be used by regulators/HTA agencies and registry holders to evaluate the applicability in HTA [66]. EUnetHTA also formulated recommendations for post-launch evidence generation using high-quality registries [67]. However, we feel that there is a gap between the perspective of regulators and HTA agencies on one side and the practical implementation of academia-led registries by academics with minimal regulatory and HTA experience on the other side. This publication and more examples of multi-purpose rare disease registries, for example in the context of the Dutch program “Managing patient registries for expensive drugs’’, contribute to hands-on experience within academia. This enables the development of best practice recommendations which might be even more valuable than general guidelines on necessities of registries.

A starting point for this study was the achievement of harmonization of the selected data elements in both treated and untreated MLD patients [68]. Harmonized clinical guidelines for rare diseases are of importance, but this is becoming more important with the emergence of new disease modifying therapies. Development of an academia-led registry for MLD can support both clinical decisions making for these new treatments as well as research into the natural disease course, development of biomarkers and genetics. For this purpose, we started with consulting patients (and their caregivers where appropriate) as well as clinical and HTA experts. With future adaptations, input from more patients and (para)medics will be taken into account. With a modified Delphi study, MLD-experts of various centers and specialties joined forces and agreed on a comprehensive set of data elements, including crucial demographics, diagnostics, clinical, and treatment-related characteristics. This approach benefited multi-disciplinary collaboration and will help implementation of the MLDi registry. It also corroborates the idea that the engagement of multiple stakeholders prior to launching a registry is necessary to represent a broad range of interests and to ensure all aims are covered in the setup, including the input of regulatory and HTA bodies [16].

A distinction between minimal, core, and optional data elements was made to prioritize the minimal and core data elements. In this way, the collection of the most important data elements is emphasized and the chances of collecting those are increased.

Apart from demographics, diagnostics, clinical, and treatment-related characteristics, consensus was mainly reached on important clinical endpoints. These endpoints included also functional scoring systems established both for MLD and for related conditions (i.e., GMFC-MLD, ELFC-MLD, MACS, EDACS). These scoring systems are validated only in patients aged below 18 or 21 years [27–30], but based on the descriptive nature of those scoring systems, the expert panel suggested that their use in patients above 18/21 years will be helpful as well.

Late onset MLD, including late-juvenile and adult MLD, typically has a more heterogeneous disease course compared to pediatric MLD [5]. In particular in adult patients, various cognitive and motor disabilities have a substantial interplay and consequently, it is difficult to measure disease progression using a single scale. Since both cognitive and motor deficits result in impaired activities of daily living, a dedicated ADL scale, such as the Barthel index, may be a sensitive tool to measure progression but needs to be evaluated in longitudinal studies.

Discussions within the expert panel further focused on biomarkers. So far, biomarkers that are validated as surrogate outcomes are not available for MLD. The expert panel agreed that promising candidate biomarkers, such as neurofilament light [69], should be further investigated and validated before inclusion in the registry. The panel also discussed whether to collect neurophysiological parameters, such as nerve conduction measurements, for the evaluation of peripheral neuropathy. As methods and parameters used grossly vary across centers, inter-center comparison is challenging. Therefore, the expert panel agreed that collection of nerve conduction measurements in the registry does currently not lead to high-quality data.

PROMs are important instruments to evaluate patients’ perspectives on health-related quality of life (HRQoL) and can be used to raise engagement of patients and their families in research and healthcare [23, 63, 70]. The consulted HTA experts from the Dutch Health Care Institute emphasized the importance of HRQoL in relative effectiveness assessments for decision-making in the context of new therapeutics [71]. However, choosing the right instrument for (young) people with cognitive impairments, such as MLD patients, is challenging [72]. A complicating factor is that patients with MLD are often unable to complete PROMs themselves due to their young age and/or cognitive decline. As PROMs will therefore often be completed by parents or guardians, it is not clear whether HRQoL or parental resilience is measured. Recently, the burden of disease on families with an MLD-affected child was investigated using the PedsQL family impact module and a semi-standardized questionnaire. This study showed that parents of children with MLD had a significant lower HRQoL compared to parents of healthy children, emphasizing the need for more research in this area [55]. Validating PROMs and applying PROMs in clinical practice has been underexposed in the MLD field so far. Hence, the EQ5D as well as the PedsQL and HUI (used successfully in another leukodystrophy, Vanishing White Matter [56]) were added to the list of data elements. In the future, one of these may gain superiority and therefore become the preferred instrument.

The present sets of data elements seem to be consistent with other frameworks for lists of data elements and are in line with the recommendations of the guideline on registry-based studies published by the EMA and the set of common data elements from the European Platform on Rare Disease Registration [23–25]. In contrast to the proposed lists of data elements, stricter privacy criteria will be applied to overcome international differences in privacy and data protection legislation. This means, for example, that no full dates will be registered, and different purposes of the data will be distinguished in the informed consent procedure.

It is important to note that new scientific insights and regulatory or HTA questions may lead to revisions of the established sets of data elements in a rare disease registry. Trials or additional studies in controlled settings, for example studies that involve supportive care with input from paramedics, might lead to substantiated use or validation of new endpoints in MLD. Besides, it is expected that database infrastructures will improve, as well as data standardization. Data standardization of all data elements in a registry remains a challenge, in particular for rare diseases, because (1) often no guidelines are available, with limited harmonization in diagnostics and treatments and (2) existing ontologies are not sufficient to describe disease-specific features. Therefore, the MLDi registry will pursue continuous improvement, also after launching. As emphasized by Kodra et al. (2018) main focuses will be increasing the quality of data and the findability, accessibility, interoperability, and reusability of the data (FAIR principles) [73].

## Conclusion

The generated dataset is expected to help answer relevant research and regulatory questions within the field of MLD. It will boost research on genotype-phenotype-correlations, natural history, and identification and validation of biomarkers. This will help making decisions on treatment eligibility, and compare different treatments in terms of safety profile and effectiveness. In addition, the current approach may assist in optimizing the existing frameworks for rare disease registries.

## Supplementary Information

**Additional file 1:** Search strings. Additional information about literature review

**Additional file 2:** Flow chart. Additional information about literature review.

**Additional file 3:** Levels of evidence and Summary tables literature review. Levels of Evidence according to The Oxford Centre for Evidence-based Medicine. Level 6 was added to take the input from patients and caregivers into account. Summary table of reviewed full texts.

**Additional file 4:** Complete core and optional set of data elements. Complete lists of data elements.

## Data Availability

Not applicable.

## Definitions

Data element: Endpoint/outcome/item/variable to be collected in the MLDi registry
Set: Group of data elements
Minimal data elements: A limited number of mandatory data elements for every included patient in the MLDi registry
Core data elements: Data elements that are considered essential for the purpose of the MLDi registry
Optional data elements: Data elements that are considered of interest and useful to some stakeholders of the MLDi registry, but not essential to all
Patient-reported outcome measures (PROMs): Outcomes reported by patients or their caregivers, assessing health-related quality of life and burden of disease
Dataset: The data from the MLDi registry made available for the purpose of a (registry-based) study
Clinical reported dataset (CRD): Observational data collection of minimal, core and optional data elements
Patient reported dataset (PRD): The data from the PROMs collected in the MLDi registry
Registry-based study: A study performed with data collected in a registry
Expert panel: Child neurologists, neurologists and transplant specialists who participated in this modified Delphi procedure
Panelist: Member of expert panel
Stakeholders: Patients, patient advocacy groups, drug regulators, HTA agencies/payers, companies, and others involved with the MLDi registry
Health technology assessment (HTA): Systematic evaluation of the impact of a new health technology such as a therapy to inform decision-making in health care.
European rare disease (EU-RD) Platform: Initiative of the European Commission to cope with the fragmentation of rare disease data in registries across Europe.
European reference network on rare neurological disorders (ERN-RND): European network to connect healthcare professionals with expertise in rare neurological diseases
Controller: According to the general data protection regulation (GDPR): Natural or legal entity, public authority, agency or other body which, alone or jointly with others, determines the purposes and means of the processing of personal data
Processor: According to the GDPR: Natural or legal person, public authority or other body which processes personal data on behalf of the controller

## Declarations

### Disclaimer

PM is an employee of the Dutch Medicines Evaluation Board, and the views expressed in this article are his own and may not necessarily reflect the position of, the Medicines Evaluation Board.
