# Supplementary material for: Modified Delphi procedure-based expert consensus on endpoints for an international disease registry for Metachromatic Leukodystrophy: The European Metachromatic Leukodystrophy initiative (MLDi)
Source: Orphanet J Rare Dis. 2022 Feb 14;17:48. doi: 10.1186/s13023-022-02189-w (PMC8842918; doi:10.1186/s13023-022-02189-w)
Supplement: Supplementary file 1 — Additional file 1: Search strings. Additional information about literature review [file 13023_2022_2189_MOESM1_ESM.pdf]

Additional file 1 – Search strings

PubMed

(((((((((((((((((“Metachromatic Leukodystrophy”[Title/Abstract]) OR “Metachromatic Leucodystrophy”[Title/Abstract]) OR “ARSA Deficiency”[Title/Abstract]) OR “Arylsulfatase A Deficiency”[Title/Abstract]) OR “Cerebroside Sulfatase Deficiency”[Title/Abstract]) OR “Cerebroside 3-Sulfatase Deficiency”[Title/Abstract]) OR “Diffuse Cerebral Sclerosis”[Title/Abstract]) OR “Greenfield Disease”[Title/Abstract]) OR “Greenfield Syndrome”[Title/Abstract]) OR “Metachromatic Leukoencephaly”[Title/Abstract]) OR “Metachromatic Leukoencephalopathy”[Title/Abstract]) OR “Sulfatide Lipidosis”[Title/Abstract]) OR “Sulphatide Lipidosis”[Title/Abstract]) OR “Sulfatidosis”[Title/Abstract]) OR “Cerebroside Sulfate Storage Disease”[Title/Abstract]) OR “Cerebroside Sulphate Storage Disease”[Title/Abstract]) OR “Mckusick 25010”[Title/Abstract]) OR “Leukodystrophy, Metachromatic”[MeSH Terms]) NOT ((“animals”[MH]) NOT humans [mh]) NOT (“animal*”[Title]) NOT (("case report*”[Title/Abstract]) OR "Case Reports"[PT]) NOT ("Geograp*”[Title/Abstract]) NOT ("Mimic*”[Title/Abstract]) NOT ("leukodystrophies”[Title]) NOT (("disorders”[Title] OR "diseases"[Title]) NOT (("novel"[Title] AND "variant*”[Title]) NOT (("novel"[Title] AND "mutation*"[Title]) NOT (("spectrum"[Title] AND "mutation*"[Title]) NOT (("first"[Title] AND "mutation*"[Title]) NOT ((("Tumor*”[Title/Abstract] OR "Parkinson*”[Title/Abstract] OR "Alzheimer*”[Title/Abstract]) NOT ("reply”[Title]) NOT ("iPSC*”[Title/Abstract])

Embase

('metachromatic leukodystrophy':ti OR 'metachromatic leucodystrophy':ti OR 'arsa deficiency':ti OR 'arylsulfatase a deficiency':ti OR 'cerebroside sulfatase deficiency':ti OR 'cerebroside 3-sulfatase deficiency':ti OR 'diffuse cerebral sclerosis':ti OR 'greenfield disease':ti OR 'greenfield syndrome':ti OR 'metachromatic leukoencephaly':ti OR 'metachromatic leukoencephalopathy':ti OR 'sulfatide lipidosis':ti OR 'sulphatide lipidosis':ti OR 'sulfatidosis':ti OR 'cerebroside sulfate storage disease':ti OR 'cerebroside sulphate storage disease':ti OR 'mckusick 25010':ti OR 'metachromatic leukodystrophy'/de) AND [humans]/lim AND [english]/lim AND [2000-2020]/py AND ([article]/lim OR [article in press]/lim OR [review]/lim) AND ('case control study'/de OR 'clinical article'/de OR 'clinical protocol'/de OR 'clinical study'/de OR 'clinical trial'/de OR 'cohort analysis'/de OR 'comparative study'/de OR 'controlled clinical trial'/de OR 'controlled study'/de OR 'cross sectional study'/de OR 'family study'/de OR 'major clinical study'/de OR 'medical record review'/de OR 'multicenter study'/de OR 'observational study'/de OR 'phase 2 clinical trial'/de OR 'phase 2 clinical trial topic'/de OR 'phase 3 clinical trial topic'/de OR 'prospective study'/de OR 'retrospective study'/de OR 'systematic review'/de) NOT ('animal*':ti OR 'geograp*':ti OR 'mimic*':ti OR 'leukodystrophies':ti OR 'disorders':ti OR 'diseases':ti OR 'tumor*':ti OR 'parkinson*':ti OR 'alzheimer*':ti OR 'ipsc*':ab,ti OR 'reply':ti) NOT ('novel':ti AND 'mutation*':ti) NOT ('novel':ti AND 'variant*':ti)
