# Supplementary figures and images for: Modified Delphi procedure-based expert consensus on endpoints for an international disease registry for Metachromatic Leukodystrophy: The European Metachromatic Leukodystrophy initiative (MLDi)
Source: Orphanet J Rare Dis. 2022 Feb 14;17:48. doi: 10.1186/s13023-022-02189-w (PMC8842918; doi:10.1186/s13023-022-02189-w)

# Additional file 2 – Flow chart

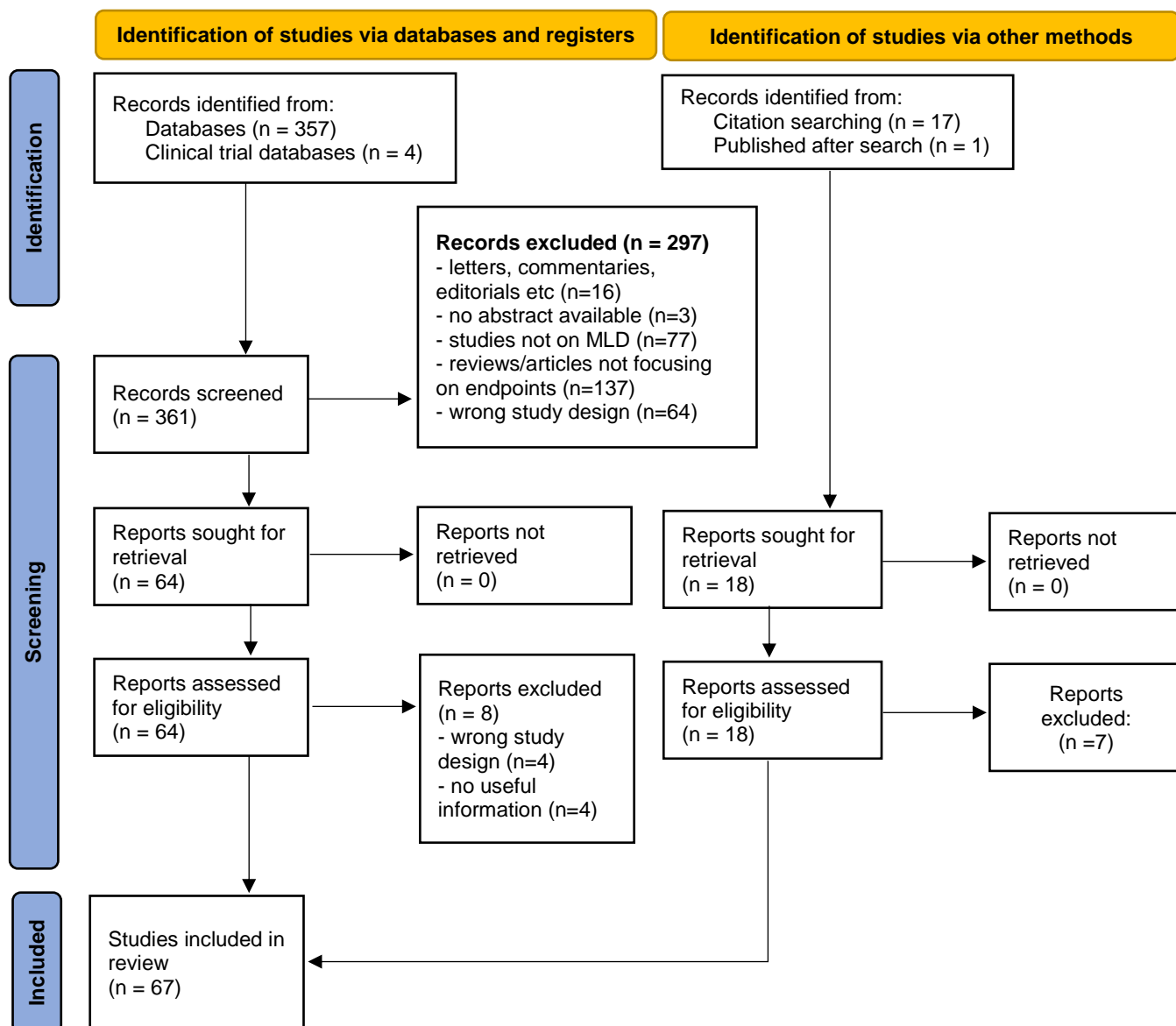

Supplement: Supplementary file 2 — Additional file 2: Flow chart. Additional information about literature review. [file 13023_2022_2189_MOESM2_ESM.pdf]
