# Supplementary material for: Modified Delphi procedure-based expert consensus on endpoints for an international disease registry for Metachromatic Leukodystrophy: The European Metachromatic Leukodystrophy initiative (MLDi)
Source: Orphanet J Rare Dis. 2022 Feb 14;17:48. doi: 10.1186/s13023-022-02189-w (PMC8842918; doi:10.1186/s13023-022-02189-w)
Supplement: Supplementary file 3 — Additional file 3: Levels of evidence and Summary tables literature review. Levels of Evidence according to The Oxford Centre for Evidence-based Medicine. Level 6 was added to take the input from patients and caregivers into account. Summary table of reviewed full texts. [file 13023_2022_2189_MOESM3_ESM.pdf]

Additional file 3 - Summary tables literature review

Supplementary TABLE 1 Levels of Evidence according to The Oxford Centre for Evidence-based Medicine.(1) Level 6 was added to take the input from patients and caregivers into account.

Level	Therapy / Prevention, Aetiology / Harm	Prognosis	Diagnosis	Differential diagnosis / symptom prevalence study	Economic and decision analyses
1a	SR (with homogeneity) of RCTs	SR (with homogeneity) of inception cohort studies; CDR validated in different populations	SR (with homogeneity) of Level 1 diagnostic studies; CDR with 1b studies from different clinical centres	SR (with homogeneity) of prospective cohort studies	SR (with homogeneity) of Level 1 economic studies
1b	Individual RCT (with narrow Confidence Interval)	Individual inception cohort study with > 80% follow-up; CDR validated in a single population	Validating cohort study with good reference standards; or CDR tested within one clinical centre	Prospective cohort study with good follow-up	Analysis based on clinically sensible costs or alternatives; systematic review(s) of the evidence; and including multi-way sensitivity analyses
1c	All or none	All or none case-series	Absolute SpPins and SnNouts	All or none case-series	Absolute better-value or worse-value analyses
2a	SR (with homogeneity) of cohort studies	SR (with homogeneity) of either retrospective cohort studies or untreated control groups in RCTs	SR (with homogeneity) of Level >2 diagnostic studies	SR (with homogeneity) of 2b and better studies	SR (with homogeneity) of Level >2 economic studies
2b	Individual cohort study (including low quality RCT; e.g., <80% follow-up)	Retrospective cohort study or follow-up of untreated control patients in an RCT; Derivation of CDR or validated on split-sample only	Exploratory cohort study with good reference standards; CDR after derivation, or validated only on split-sample or databases	Retrospective cohort study, or poor follow-up	Analysis based on clinically sensible costs or alternatives; limited review(s) of the evidence, or single studies; and including multi-way sensitivity analyses
2c	"Outcomes" Research; Ecological studies	"Outcomes" Research		Ecological studies	Audit or outcomes research
3a	SR (with homogeneity) of case-control studies		SR (with homogeneity) of 3b and better studies	SR (with homogeneity) of 3b and better studies	SR (with homogeneity) of 3b and better studies

3b	Individual Case-Control Study		Non-consecutive study; or without consistently applied reference standards	Non-consecutive cohort study, or very limited population	Analysis based on limited alternatives or costs, poor quality estimates of data, but including sensitivity analyses incorporating clinically sensible variations.
4	Case-series (and poor quality cohort and case-control studies)	Case-series (and poor quality prognostic cohort studies)	Case-control study, poor or non-independent reference standard	Case-series or superseded reference standards	Analysis with no sensitivity analysis
5	Expert opinion without explicit critical appraisal, or based on physiology, bench research or "first principles"	Expert opinion without explicit critical appraisal, or based on physiology, bench research or "first principles"	Expert opinion without explicit critical appraisal, or based on physiology, bench research or "first principles"	Expert opinion without explicit critical appraisal, or based on physiology, bench research or "first principles"	Expert opinion without explicit critical appraisal, or based on economic theory or "first principles"
6	Qualitative research	Qualitative research	Qualitative research	Qualitative research	Qualitative research
-	Inconclusive	Inconclusive	Inconclusive	Inconclusive	Inconclusive

Abbreviations: CDR = Clinical Decision Rule; RCT = randomized controlled trial; SpPins and SnNouts = 'An "Absolute SpPin" is a diagnostic finding whose Specificity is so high that a Positive result rules-in the diagnosis. An "Absolute SnNout" is a diagnostic finding whose Sensitivity is so high that a Negative result rules-out the diagnosis'; SR = Systematic Review.

1. Bob Phillips CB DS, Doug Badenoch, Sharon Straus, Brian Haynes, Martin Dawes Oxford Centre for Evidence-based Medicine – Levels of Evidence 2009 [updated since November 1998, Updated by Jeremy Howick March 2009. Available from: https://www.cebm.net/2009/06/oxford-centre-evidence-based-medicine-levels-evidence-march-2009/2009.

Supplementary TABLE 2 Summary table of reviewed full texts

author, year	Study design	Level of evidence	Patients (n =)	Controls (n =)	MLD TYPE	Main DATA ELEMENTS (s)	HSCT/GT/ ERT	Country	Full citation
Artigalás, O., 2010	Cross-sectional retrospective	3b	29		Late infantile (n=22), juvenile (n=4), adult (n=1)	Baseline patient<br>Baseline disease<br>Motor signs<br>Cognition and behaviour<br>MRI<br>Neurophysiology	Untreated	Brazil	Artigalás, O., et al. (2010). "Clinical and biochemical study of 29 Brazilian patients with metachromatic leukodystrophy." J Inherit Metab Dis 33 Suppl 3: S257-262.
Assadi, M., 2013	Case-control study	4	4*		Late infantile	Magnetic resonance spectroscopy	Untreated	USA	Assadi, M., et al. (2013). "Multi-Voxel 1H-MRS in Metachromatic Leukodystrophy." J Cent Nerv Syst Dis 5: 25-30.
Assadi, M.,2012	Open-labeled prospective trial (pilot)	4	4*		Late infantile	Magnetic resonance spectroscopy	Untreated	USA	Assadi, M., et al. (2012). "Vitamin k antagonist warfarin for palliative treatment of metachromatic leukodystrophy, a compass-sionate study of four subjects." J Cent Nerv Syst Dis 4: 73-79.
Baumann, N., 2002	cross-sectional retrospective	4	12		Adult	Genotype-phenotype correlation<br>Motor signs<br>Cognition and behaviour<br>Other clinical signs	Untreated	France	Baumann, N., et al. (2002). "Motor and psycho-cognitive clinical types in adult metachromatic leukodystrophy: Genotype/phenotype relationships?" Journal of Physiology Paris 96(3-4): 301-306.
Beerepoot, S., 2020	Retrospective cross-section	4	76		late infantile (n=11), juvenile (n=39), adult (n=17)	Genotype-phenotype correlation		The Netherlands	Beerepoot S, van Dooren SJM, Salomons GS, et al. Metachromatic leukodystrophy genotypes in The Netherlands reveal novel pathogenic ARSA variants in non-Caucasian patients [published online ahead of print, 2020 Jul 7]. Neurogenetics. 2020;10.1007/s10048-020-00621-

									6. doi:10.1007/s10048-020-00621-6
Beerepoot, S., 2019	Literature review	-				Peripheral neuropathy		The Netherlands	Beerepoot, S., et al. (2019). "Peripheral neuropathy in metachromatic leukodystrophy: current status and future perspective." Orphanet J Rare Dis 14(1): 240.
Biffi, A. 2008	Cross-sectional	2b	26		Late infantile (n=16), early-juvenile (n=7), late-juvenile (n=2), adult (1)	Genotype-phenotype correlation	Untreated	Italy	Biffi, A., et al. (2008). "Metachromatic leukodystrophy - mutation analysis provides further evidence of genotype-phenotype correlation." Clin Genet 74(4): 349-357.
Biffi, A. 2013	Clinical trial	4	3		Late infantile	HSCT-GT	GT	Italy	Biffi A, Montini E, Lorioli L, Cesani M, Fumagalli F, Plati T, et al. Lentiviral hematopoietic stem cell gene therapy benefits metachromatic leukodystrophy. Science (New York, NY). 2013;341(6148).
Bindu, P. S., 2005	Single-centre cohort	2b	40		Late infantile (n=36), juvenile (n=4)	Baseline patient<br>Baseline disease<br>Motor signs<br>Cognition and behaviour<br>MRI<br>Neurophysiology<br>Pathology	Untreated	India	Bindu, P. S., et al. (2005). "Peripheral neuropathy in metachromatic leucodystrophy. A study of 40 cases from south India." J Neurol Neurosurg Psychiatry 76(12): 1698-1701.
Bonkowsky, J. L., 2018	Case-control study, retrospective	-	(557) 139 MLD		?	Race/ethnicity	?	USA	Bonkowsky, J. L., et al. (2018). "Association of Diagnosis of Leukodystrophy With Race and Ethnicity Among Pediatric and Adolescent Patients." JAMA Netw Open 1(7): e185031.

Boucher, A. A. 2015	Single-centre cohort, retrospective	2c	40		Late infantile (n=4), juvenile (n=27), adult (n=9)	HSCT related	HSCT	USA	Boucher, A. A., et al. (2015). "Long-term outcomes after allogeneic hematopoietic stem cell transplantation for metachromatic leukodystrophy: the largest single-institution cohort report." Orphanet J Rare Dis 10: 94.
Brown, T. M., 2017	Literature review and qualitative	2b	5		Juvenile	Quality of life and impact of disease	untreated	USA	Brown, T. M., et al. (2017). "Development of the Impact of Juvenile Metachromatic Leukodystrophy on Physical Activities scale." J Patient Rep Outcomes 2(1): 15.
Cameron, C. L., 2004	Retrospective cohort	3b	9		Late infantile or early juvenile	Neurophysiology	?	USA	Cameron, C. L., et al. (2004). "Multifocal slowing of nerve conduction in metachromatic leukodystrophy." Muscle and Nerve 29(4): 531-536.
Cesani, M., 2016	Systematic review	2a				Genotype<br>Genotype-phenotype		Italy	Cesani M, Lorioli L, Grossi S, Amico G, Fumagalli F, Spiga I, et al. Mutation Update of ARSA and PSAP Genes Causing Metachromatic Leukodystrophy. Hum Mutat. 2016;37(1):16-27.
Chen, X., 2016	Case-control with untreated siblings	3b	3	2	Early juvenile	UCBT related	UBCT	China	Chen, X., et al. (2016). "Outcome of Early Juvenile Onset Metachromatic Leukodystrophy after Unrelated Cord Blood Transplantation." Journal of Child Neurology 31(3): 338-344.
Clas, P., 2012	reproduction/ validation study	1b	35		.	MRI	Untreated	Germany	Clas, P., et al. (2012). "A semi-automatic algorithm for determining the demyelination load in metachromatic leukodystrophy." Acad Radiol 19(1): 26-34.

Dali, C., 2010	Cross-section	2b	13**		Late infantile	Magnetic Resonance Spectroscopy	Untreated	Denmark	Dali, C., et al. (2010). "Brain N-acetylaspartate levels correlate with motor function in metachromatic leukodystrophy." Neurology 75(21): 1896-1903.
Dali, CÍ, 2015	Cross-section	2b	13**		Late-infantile	Neurophysiology<br>Magnetic Resonance Spectroscopy<br>Lab results	Untreated	Denmark	Dali, C., et al. (2015). "Sulfatide levels correlate with severity of neuropathy in metachromatic leukodystrophy." Ann Clin Transl Neurol 2(5): 518-533.
Dali, C, 2020	Clinical trial, multi-center non-randomized open label phase 1/2 trial	3b	24		Late-infantile	Safety and tolerability of intrathecal administration of rhASA<br>Neurophysiology<br>Lab results	ERT	Denmark/France	Dali C, Sevin C, Krageloh-Mann I, Giugliani R, Sakai N, Wu J, et al. Safety of intrathecal delivery of recombinant human arylsulfatase A in children with metachromatic leukodystrophy: Results from a phase 1/2 clinical trial. Mol Genet Metab. 2020;131(1-2):235-44.
De Hosson, L. D., 2011	Cohort, prospective?	4	5		Adult	HSCT related	Allo-SCT	The Netherlands	De Hosson, L. D., et al. (2011). "Adult metachromatic leukodystrophy treated by allo-SCT and a review of the literature." Bone Marrow Transplantation 46(8): 1071-1076.
Eichler, F. S., 2016	Qualitative study, multi-centre	-	30 caregivers of 23 patients		Late-infantile (n=19, juvenile (n=7), adult (n=4)	Quality of life and impact of disease	.	USA	Eichler, F. S., et al. (2016). "Metachromatic Leukodystrophy: An Assessment of Disease Burden." J Child Neurol 31(13): 1457-1463.
Eichler, F., 2009	Cross-section	1b	28		Late infantile (n=10), Juvenile (n=16), adult (n=2)	MRI	.	Germany	Eichler, F., et al. (2009). "Metachromatic leukodystrophy: a scoring system for brain MR imaging observations." AJNR Am J Neuroradiol 30(10): 1893-1897.

Elgün, S., 2019	Retrospective cross-section	2b	12 sibling-pairs and 61 single patients		sibling-pairs (3 late-infantile, 9 juvenile), single patients (29 late-infantile, 32 juvenile)	Genotype-phenotype correlation	Untreated	Germany, The Netherlands	Elgün, S., et al. (2019). "Phenotypic variation between siblings with Metachromatic Leukodystrophy." Orphanet Journal of Rare Diseases 14(1).
Groeschel, S., 2011	Longitudinal cross-section	2b	68		Late infantile (n=33), juvenile (n=35)	MRI	.	Germany	Groeschel, S., et al. (2011). "Metachromatic leukodystrophy: natural course of cerebral MRI changes in relation to clinical course." J Inherit Metab Dis 34(5): 1095-1102.
Groeschel, S., 2012	Cross-section, case-control	4	18**	42	Late infantile	MRI	.	Germany	Groeschel, S., et al. (2012). "Cerebral gray and white matter changes and clinical course in metachromatic leukodystrophy." Neurology 79(16): 1662-1670.
Groeschel, S., 2016	Case-control , retrospective	3b	24	41	juvenile	HSCT related	HSCT	Germany	Groeschel, S., et al. (2016). "Long-term Outcome of Allogeneic Hematopoietic Stem Cell Transplantation in Patients With Juvenile Metachromatic Leukodystrophy Compared With Nontransplanted Control Patients." JAMA Neurol 73(9): 1133-1140.
Harrington, M., 2019	Qualitative study	-	32 caregivers		late infantile (n=16), juvenile (n=16)	Quality of life and impact of disease	3 SCT	USA	Harrington, M., et al. (2019). "Insights into the natural history of metachromatic leukodystrophy from interviews with caregivers." Orphanet J Rare Dis 14(1): 89.
Jabbehdari, S., 2015	Observational cross section	3b	18		Late infantile (n=12), juvenile (n=6)	Baseline patient<br>Baseline disease<br>Cognition<br>MRI	Untreated	Iran	Jabbehdari, S., et al. (2015). "The clinical features and diagnosis of Metachromatic leukodystrophy: A case series of Iranian Pediatric

									Patients." Iran J Child Neurol 9(3): 57-61.
Kehrer, C. 2011	Prospective and retrospective cohort	1b	59***		late infantile (n=21), juvenile (n=38)	Motor signs	Untreated	Germany	Kehrer, C., et al. (2011). "Development and reliability of a classification system for gross motor function in children with metachromatic leucodystrophy." Dev Med Child Neurol 53(2): 156-160.
Kehrer, C., 2011	Prospective and retrospective cohort	2b	59***		late infantile (n=21), juvenile (n=38)	Motor signs	Untreated	Germany	Kehrer, C., et al. (2011). "The natural course of gross motor deterioration in metachromatic leukodystrophy." Dev Med Child Neurol 53(9): 850-855.
Kehrer, C., 2014	Prospective and retrospective cohort	2b	59***		late infantile (n=21), juvenile (n=38)	Language<br>Cognition	Untreated	Germany	Kehrer, C., et al. (2014). "Language and cognition in children with metachromatic leukodystrophy: onset and natural course in a nationwide cohort." Orphanet J Rare Dis 9: 18.
Kim TS, 1997	Retrospective cross-section	4	7		Late infantile	MRI	Untreated	Korea	Kim TS, Kim I-O, Kim WS et al. MR in childhood metachromaticleukodystrophy. AmJNeuroradiol1997:18:733–738.
Kim, J., 2017	Retrospective cohort	2b	29 MLD, ALD/Krabbe: 58	.	?	Gall bladder related	HSCT	USA	Kim, J., et al. (2017). "Gallbladder abnormalities in children with metachromatic leukodystrophy." J Surg Res 208: 187-191.
Koç, O. N., 2002	Clinical trial	4	6		?	Mesenchymal stem cell related	HSCT	USA	Koç, O. N., et al. (2002). "Allogeneic mesenchymal stem cell infusion for treatment of metachromatic leukodystrophy (MLD) and Hurler syndrome (MPS-IH)." Bone Marrow Transplant 30(4): 215-222.
Krägeloh-Mann, I., 2013	Case-control	3b	2	67	Juvenile	HSCT related	HSCT	Germany	Krägeloh-Mann, I., et al. (2013). "Juvenile metachromatic

									leukodystrophy 10 years post transplant compared with a non-transplanted cohort." Bone Marrow Transplantation 48(3): 369-375.
Lorioli, L., 2015	Retrospective cohort	2b	24		Late infantile (n=13), early-juvenile (11/24)	Lab results (metabolic acidosis)	HSC-GT	Italy	Lorioli, L., et al. (2015). "Abnormalities of acid-base balance and predisposition to metabolic acidosis in Metachromatic Leukodystrophy patients." Molecular Genetics and Metabolism 115(1): 48-52.
Lugowska, A., 1997	Diagnostic method study	1b	49	30		Lab results	.	Poland	Ługowska, A., Tylki-Szymańska, A., Berger, J., & Molzer, B. (1997). Elevated sulfatide excretion in compound heterozygotes of metachromatic leukodystrophy and ASA-pseudodeficiency allele. Clinical Biochemistry, 30(4), 325–331. https://doi.org/10.1016/S0009-9120(97)00033-7
Lugowska, A., 2005	Cross-section	2b	43		Late infantile (n=14), early juvenile (n=11), late-juvenile (n=7), adult (n=11)	Genotype-phenotype correlation	.	Poland	Ługowska, A., et al. (2005). "Molecular and phenotypic characteristics of metachromatic leukodystrophy patients from Poland." Clin Genet 68(1): 48-54.
Mahmood, A.,2010	Case report and systematic review	4	3		Late infantile	Survival<br>Natural disease course	.	USA	Mahmood, A., et al. (2010). "Metachromatic leukodystrophy: A case of triplets with the late infantile variant and a systematic review of the literature." Journal of Child Neurology 25(5): 572-580.
Malcolm, C.,2011	Qualitative	-				Quality of life and impact of disease		UK	Malcolm, C., et al. (2011). "Challenging symptom profiles of

									life-limiting conditions in children: a survey of care professionals and families." Palliat Med 25(4): 357-364.
Martin, A, 2006	Clinical trial, phase 2 multi-centre	4	69 (6 MLD)			UCBT related	UBCT	USA	Martin, P. L., Carter, S. L., Kernan, N. A., Sahdev, I., Wall, D., Pietryga, D., … Kurtzberg, J. (2006). Results of the Cord Blood Transplantation Study (COBLT): Outcomes of unrelated donor umbilical cord blood transplantation in pediatric patients with lysosomal and peroxisomal storage diseases. Biology of Blood and Marrow Transplantation, 12(2), 184–194. https://doi.org/10.1016/j.bbmt.2005.09.016
Martin, A., 2012	Retrospective case-control	4	13		Late infantile, early juvenile	MRI<br>Magnetic Resonance Spectroscopy	.	France	Martin, A., et al. (2012). "Toward a better understanding of brain lesions during metachromatic leukodystrophy evolution." AJNR Am J Neuroradiol 33(9): 1731-1739.
Martin, A., 2013	Retrospective cohort	2b	27		Late infantile (n=10), juvenile (n=17)	UCBT related	HSCT	USA	Martin, H. R., et al. (2013). "Neurodevelopmental outcomes of umbilical cord blood transplantation in metachromatic leukodystrophy." Biol Blood Marrow Transplant 19(4): 616-624.
Martin, HR 2008	Retrospective review	4				Cognition			Martin HR, Poe MD, Reinhartsen D, et al. Methods for assessing neurodevelopment in lysosomal storage diseases and related disorders:<br>a multi-disciplinary perspective.

									Acta Paediatr. 2008;97(Suppl.): 69-75.
Parikh s., 2015	Systematic review	3a			Leukodystrop hies	Clinical diagnostics		USA	Parikh S, Bernard G, Leventer RJ, van der Knaap MS, van Hove J, Pizzino A, et al. A clinical approach to the diagnosis of patients with leukodystrophies and genetic leukoencephelopathies. Mol Genet Metab. 2015;114(4):501-15
Raina, A., 2019	Retrospective cross section	3b	12		Juvenile (n=8), late infantile (n=4)	Neurophysiology<br>MRI	untreated?	India	Raina, A., et al. (2019). "Electroneurography and advanced neuroimaging profile in pediatric-onset metachromatic leukodystrophy." Journal of Pediatric Neurosciences 14(2): 70-75.
Saute, J. A. M., 2016	Retrospective cohort	4	2			HSCT related	HSCT	Brazil	Saute, J. A. M., et al. (2016). "Neurological outcomes after hematopoietic stem cell transplantation for cerebral X-linked adrenoleukodystrophy, late onset metachromatic leukodystrophy and hurler syndrome." Arquivos de Neuro-Psiquiatria 74(12): 953-966.
Sessa, M., 2016	Non-randomised, open label, phase 1/2 trial	3b	9	historical untreated controls	Late- infantile (n=6), early-juvenile (n=2), undefined (n=1)	HSCT-GT related	HSC-GT	Italy	Sessa, M., et al. (2016). "Lentiviral haemopoietic stem-cell gene therapy in early-onset metachromatic leukodystrophy: an ad-hoc analysis of a non-randomised, open-label, phase 1/2 trial." Lancet 388(10043): 476-487.
Sevin C, 2018	Clinical trial	4	4		Late infantile (n=3), juvenile (n=1)	Intracerebral GT	Intracerebral GT	France	Sevin C, Roujeau T, Cartier N, et al. Intracerebral gene therapy in children with metachromatic leukodystrophy: Results of a phase

									I/II trial. Molecular Genetics and Metabolism 2018;123(2):S129-S29. doi: 10.1016/j.ymgme.2017.12.352
Solders, M., 2014	Case control	3b	4	3	Adult	HSCT related	HSCT	Sweden	Solders, M., et al. (2014). "Hematopoietic SCT: a useful treatment for late metachromatic leukodystrophy." Bone Marrow Transplant 49(8): 1046-1051.
Strölin, M., 2017	Retrospective cohort	2b	46		Juvenile	MRI	.	Germany	Strölin, M., et al. (2017). "Demyelination load as predictor for disease progression in juvenile metachromatic leukodystrophy." Ann Clin Transl Neurol 4(6): 403-410.
Tan, m.a., 2010	Validation study	1b	11	18	Late infantile (n=5), juvenile (n=5), adult (n=3)	Biochemical diagnosis		Australia	Tan MA, Fuller M, Zabidi-Hussin ZA, Hopwood JJ, Meikle PJ. Biochemical profiling to predict disease severity in metachromatic leukodystrophy. Mol Genet Metab. 2010;99(2):142-8.
Thibert, K. A., 2016	Case control	4	8		Late infantile (n=1), early juvenile (n=2), late juvenile (n=2), adult (n=3)	Lab results	.	USA	Thibert, K. A., et al. (2016). "Cerebral Spinal Fluid levels of Cytokines are elevated in Patients with Metachromatic Leukodystrophy." Sci Rep 6: 24579.
Tillema, J. M.,2015	Retrospective case-control	2b	20	20	Late infantile (n=3), juvenile (n=11), adult (n=6)	MRI	Untreated	The Netherlands	Tillema, J. M., et al. (2015). "Volumetric MRI data correlate to disease severity in metachromatic leukodystrophy." Ann Clin Transl Neurol 2(9): 932-940.
Toldo, I. 2005	Case report	5	1		Late infantile (n=1)	Spinal cord		Germany	Toldo I, Carollo C, Battistella PA, Laverda AM. Spinal cord and cauda equina MRI findings in metachromatic leukodystrophy:

									case report. Neuroradiology. 2005;47(8):572-5.
van der Veldt, N., 2019	Case series + case control	3b	10	8 (CP patients)	Early juvenile (n-3), late juvenile (n=7)	Motor signs<br>Intrathecal Baclofen related	Untreated	The Netherlands	van der Veldt, N., et al. (2019). "Intrathecal baclofen in metachromatic leukodystrophy." Developmental Medicine and Child Neurology 61(2): 232-235.
van rappard, d.f., 2015	Systematic review	3a				Clinical diagnosis<br>Clinical spectrum<br>Treatment approaches		The Netherlands	van Rappard DF, Boelens JJ, Wolf NI. Metachromatic leukodystrophy: Disease spectrum and approaches for treatment. Best practice & research Clinical endocrinology & metabolism. 2015;29(2):261-73.
van Rappard, D. F., 2016	Retrospective cohort	3b	13	22 (untreated)	Late infantile (n=8), Juvenile (n=18), adult (n=9)	HSCT related	HSCT	The Netherlands	van Rappard, D. F., et al. (2016). "Efficacy of hematopoietic cell transplantation in metachromatic leukodystrophy: the Dutch experience." Blood 127(24): 3098-3101.
Van Rappard, D. F., 2016	Retrospective cohort	3b	34		Late infantile (n=4), juvenile (n=22), adult (n=9)	Gall bladder related	14/34 HSCT	The Netherlands	Van Rappard, D. F., et al. (2016). "Gallbladder and the risk of polyps and carcinoma in metachromatic leukodystrophy." Neurology 87(1): 103-111.
van Rappard, D. F., 2018	Case series	4	4			Psychiatric signs	2/4 HSCT	The Netherlands	van Rappard, D. F., et al. (2018). "Slowly Progressive Psychiatric Symptoms: Think Metachromatic Leukodystrophy." J Am Acad Child Adolesc Psychiatry 57(2): 74-76.
van Rappard, D. F., 2018	Retrospective cohort	1b	21	16	Juvenile (n=12), adult (n=9)	Magnetic Resonance Spectroscopy	12/21 HSCT	The Netherlands	van Rappard, D. F., et al. (2018). "Quantitative MR spectroscopic imaging in metachromatic leukodystrophy: value for prognosis and treatment." J Neurol Neurosurg Psychiatry 89(1): 105-111.

Wadhwa, A., 2019	Retrospective cohort	2b	273 (10.2% MLD)			HSCT related	HSCT (BM, CB)	USA	Wadhwa, A., et al. (2019). "Late Mortality after Allogeneic Blood or Marrow Transplantation for Inborn Errors of Metabolism: A Report from the Blood or Marrow Transplant Survivor Study-2 (BMTSS-2)." Biol Blood Marrow Transplant 25(2): 328-334
Yavuz, H., 2011	Case report	5	1		Late infantile	Abdominal symptoms	Untreated	Turkey	Yavuz, H. and H. A. Yüksekkaya (2011). "Intestinal involvement in metachromatic leukodystrophy." J Child Neurol 26(1): 117-120.

<u>Legend</u>

Level of evidence according to Supplementary table 3.

* same cohort

** (partly) same cohort

*** same cohort

Supplementary TABLE 3 Summary table of reviewed trial protocols in literature review

Trial identification no.		Trial description
NCT01510028	Clinical trial	Multicenter Study of HGT-1110 Administered Intrathecally in Children With Metachromatic Leukodystrophy (MLD)
NCT01560182	Clinical trial	This Phase I/II clinical trial consists of the application of lentiviral vector-based gene therapy to patients affected by Metachromatic Leukodystrophy (MLD), a rare inherited Lysosomal Storage Disorder (LSD) resulting from mutations in the gene encoding the Arylsulfatase A (ARSA) enzyme. The medicinal product consists of autologous CD34+ hematopoietic stem/progenitor cells in which a functional ARSA cDNA is introduced by means of 3rd generation VSV-G pseudotyped lentiviral vectors.
NCT02559830	Clinical trial	Evaluating the safety and efficacy of Lentiviral Hematopoietic Stem Cell Gene Therapy for advanced stage of Metachromatic Leukodystrophy and adrenoleukodystrophy.
NCT03392987	Clinical trial	OTL-200 is autologous CD34+ cells transduced with lentiviral vector containing human arylsulfatase A (ARSA) complementary deoxyribonucleic acid (cDNA) used for the treatment of MLD. MLD is an autosomal recessive lysosomal storage disorder (LSD) characterized by severe and progressive demyelination affecting the central and peripheral nervous system. This study will assess safety and efficacy of treatment using cryopreserved formulation of OTL-200 in pediatric subjects with pre-symptomatic Early Onset MLD (Late Infantile (LI) to Early Juvenile (EJ) MLD) and early symptomatic EJ MLD.
