# Supplementary material for: Modified Delphi procedure-based expert consensus on endpoints for an international disease registry for Metachromatic Leukodystrophy: The European Metachromatic Leukodystrophy initiative (MLDi)
Source: Orphanet J Rare Dis. 2022 Feb 14;17:48. doi: 10.1186/s13023-022-02189-w (PMC8842918; doi:10.1186/s13023-022-02189-w)
Supplement: Supplementary file 4 — Additional file 4: Complete core and optional set of data elements. Complete lists of data elements. [file 13023_2022_2189_MOESM4_ESM.pdf]

Additional file 4 – Complete core and optional set of data elements

Supplementary TABLE 4 Core set of data elements

Core set

**Data element**	**Coding**
Ethnic background	According to the United Nations Geoscheme<br>Northern Africa, Sub-Saharan Africa, Eastern Africa, Middle Africa, Southern Africa, Western Africa, Caribbean, Central America, South America, Northern America, Central Asia, Eastern Asia, South-eastern Asia, Southern Asia, Western Asia, Eastern Europe (including Northern Asia), Northern Europe, Southern Europe, Western Europe
Country of residence	Countries
Significant family history	No relevant family history, Parkinson's disease, other, unknown
Sibling with MLD	Yes, no, unknown<br>Record ID of sibling
Consent to future sharing of encoded data with physicians abroad through the MLD initiative	Yes, no, unknown
Consent to future sharing of encoded data with researchers outside the European Union	Yes, no, unknown
Consent to future sharing of encoded data with companies developing new treatments for MLD and with (semi)governmental bodies involved in making healthcare policies.	Yes, no, unknown
Clinical trial participation	Yes (>specify), no, unknown
MLD type	late-infantile (<2.5 years), early-juvenile (2.5 - 6 years), late-juvenile (6 - 16 years), adult (>16 years)
Both genetic mutations	• c.256C>T, p.(Arg86Trp)<br>• c.257G>A, p.(Arg86Gln)<br>• c.293C>T, p.(Ser98Phe)<br>• c.465+1G>A<br>• c.542T>G, p.(Ile181Ser)<br>• c.1210+1G>A<br>• c.1283C>T, p.(Pro428Leu)<br>• Specify other ARSA mutation<br>• Specify PSAP mutation<br>• Specify SUMF1 mutation<br>• Presence of pseudodeficiency allele (c.1055A > G, p.(Asn352Ser) + *96A > G)
ASA activity in leukocytes or fibroblasts at diagnosis	Leukocytes/Fibroblasts > in nmol/mg protein/h / nmol/17hmg protein / % from mean reference value
Urinary sulfatide level	In nmol/mmol creatinine

Symptomatic at diagnosis 1	Which situation gave rise to the diagnosis: manifested symptoms, family screening because of affected sibling, newborn screening, other, unknown.
Symptomatic at diagnosis 2 & presenting symptoms	No symptoms at all, Gross motor signs (unsteady gait, clumsiness), signs of polyneuropathy, balance and coordination problems, deterioration in school performance, cognitive decline, language regression, behavioral changes, psychiatric signs, unknown, other.
Date of first contact with specialized center	mm/yyyy
Gross Motor Function Classification (GMFC-MLD)	Scale from level I - level VI<br>date of transition to other level
Expressive Language Function Classification (ELFC-MLD)	Scale from level I - level IV<br>date of transition to other level
Eating and Drinking Ability Classification System (EDACS)	Scale from level I - level V<br>date of transition to other level
Manual Ability Classification System (MACS)	Scale from level I - level V<br>date of transition to other level
Weight	Kg
Standing height, sitting height or length	m
Developmental milestones	Sitting without support, Hands-and-knees crawling, Standing with assistance, Walking with assistance, Standing alone, Walking alone (according to WHO motor development milestones)<br><br>Exact age in months or Normal development/delayed development/unknown
Cognitive decline	Yes, no, unknown
Memory problems	Yes, no, unknown
Intelligence – IQ scores<br>Total IQ score (TIQ)<br>Verbal comprehension (VIQ)<br>Perceptual Reasoning Index (PIQ)<br>Processing speed<br>Working memory	0-150
Intelligence - IQ scale used for testing	Used test: Wechsler Preschool and Primary Scale of Intelligence, Wechsler Adult Intelligence Scale–Third Edition, Wechsler intelligence Scale for Children, Dutch Groninger Intelligence Test, Bayley Scales of Infant Development, Kaufman Assessment Battery for Children, Leiter International Performance Scale, Wechsler Nonverbal Scale of Ability, Mullen Scales for Early Learning, Bourdon-Wiersema test, Wechsler memory scale-revised
Psychiatric symptoms	Yes, no, unknown<br>>Psychotic symptoms, affective disorders, behavioral disorders (>impulsive/disinhibition, aggression, inappropriate sexual behavior, apathy, ADHD, autism spectrum disorder), anxiety disorder, obsessive-compulsive disorder, change of personality
Irritability	Always, mostly, rarely, never

EuroQol 5D	PROM
Seizures	No, partial seizures only, primarily or secondarily generalized seizures
MLD Loes score	Scale from 0 - 34
Permanent tube feeding	Yes, no, unknown
Causal treatment	
Causal treatment	Yes, no, decision for causal treatment not yet made, unknown<br>Hematopoietic stem cell transplantation, Autologous hematopoietic stem cell-based gene therapy (Libmeldy^{TM}), Intrathecal enzyme replacement therapy (SHP611/HGT-1110/rhASA), other
Date of treatment	Dd/mm/yyyy<br>In case of HSCT and GT: choose date of stem cell product infusion.<br>In case of ERT: choose date of first enzyme infusion.
Symptoms at time of the treatment	Pre-symptomatic, early-symptomatic, symptomatic
Adverse events	Dd/mm/yyyy<br>Common Terminology Criteria for Adverse Events version 5.0<br><br>In addition to CTCAE:<br>acute GvHD + grade (overall grade: 1 to 4), chronic GvHD (mild, moderate or severe: NIH criteria), graft failure (autologous reconstitution recipient >95%, aplasia), neurological deterioration (disease progression), immunoglobulins against arylsulfatase A, presence of replication competent virus, pump infection, catheter disconnection<br><br>Pregnancy after transplant (unknown, no, yes, natural, preserved)
**ERT-related**	
Intrathecal enzyme replacement therapy	Dose (150mg weekly, other…)
HSCT-related	
Conditioning regimen	Bu/Cy or FluBu or CloFluBu or TreoFluTT, other (free text) + dose<br>If Bu (busulfan): what was Busulfan area under the curve (AUC)? (Free text)
Serotherapy	rATG (Sanofil), rATG (neovi), hATG, Alemtuzumab, None, unknown
Number of infused CD34+ cells per kg	Infused number x 10^6/kg
Total number of infused cells per kg	Number
Time to engraftment neutrophils<br>Defined as: Neutrophil engraftment is defined as three consecutive days where the neutrophil count is 500 cells/mm3 or more.	Number of days
Time to platelet engraftment	Number of days

Defined as: Choose the first day of the 7 consecutive days of platelets exceeding 20 x 10^9/L	
Full chimerism in blood (>95%)	>95%, 50-95%, 10-50%, unknown
Matching for HLA-A, HLA-B, and HLA-DRB1 alleles	10/10, 9/10, 8/10, 7/10, ≤6/10<br>OR for 8 antigens: 8/8, 7/8, 6/8, 5/8, or ≤4/8<br>OR for 6 antigens: 6/6,5/6, 4/6, ≤3/6
Graft-versus-Host-Disease prophylaxis	Ciclosporine, Methotrexate, mycophenolate mofetil (MMF), Tacrolimus, Sirolimus, prednisone, other (Free text),
Donor type	Unrelated, related carrier, related non-carrier, related carrier status unknown
Graft source	Bone marrow, peripheral blood, umbilical cord blood (expended cord blood)
ASA activity in leukocytes every 12 months	In nmol/mg protein/h / nmol/17hmg protein / % from mean reference value
Chimerism in blood at last follow-up	%
Test used to measure chimerism	FISH, PCR, VRTR, next generation sequencing, other
Second treatment	Yes, no, unknown
GT-related	
Conditioning regimen	Busulfan (including area under the curve)<br>What was Busulfan AUC? (Free text)
Number of infused CD34+ cells per kg	Infused number x 10^6/kg
Total number of infused cells per kg	Number x 10^6/kg
Time to engraftment neutrophils<br>Defined as: Neutrophil engraftment is defined as three consecutive days where the neutrophil count is 500 cells/mm3 or more.	Number of days
Time to platelet engraftment<br>Defined as: Choose the first day of the 7 consecutive days of platelets exceeding 20 x 10^9/L	Number of days
ASA activity in leukocytes every 12 months	In nmol/mg protein/h / nmol/17hmg protein / % from mean reference value
Second treatment	Yes, no , unknown
Used viral vector	Lentiviral, other
Vector Copy Number (VCN) per cell	Number<br>Peripheral blood/bone marrow/unknown
Transduced (LV+) cell engraftment	%
Time to transduced LV+ cell engraftment	Number of days

Supplementary TABLE 5 Optional set of data elements

Optional set

**Data element**	**Coding**
Gross Motor Function Measure-88 (GMFM-88)	%
Head circumference	cm
Puberty development	Normal, delayed, premature, not applicable
Urinary continence	Diurnal continence, nocturnal continence, unknown
Urinary catheterization	No, intermittent, permanent
Cranial nerves	Eye movements (intact/not intact) > onset symptom (yes/no) > strabismus (paralytic/concomitant), nystagmus, oculomotor apraxia, hearing
Muscle tone	Spasticity, Rigidity, Dystonia, Paratonia, none, unknown
Extrapyramidal symptoms	Chorea, tremor (resting, postural, intention), tics, myoclonus, hemiballismus
Tendon reflexes	Normal, exaggerated upper/lower extremities, absent upper/lower extremities, unknown
Plantar responses left and right	Flexor, Babinski sign, indifferent
Gait	Normal, broad-based, spastic, foot drop, unable to walk
Ataxia	Yes, no, unknown
Trunk balance	Yes, no, unknown
Frequent falls	Yes, no, unknown
Mini-mental state examination (MMSE)	Score from 0-30
School career	None, early childhood education (preschool, kindergarten), primary education (primary/elementary school), secondary education (secondary/high school), tertiary education (higher/vocational education, academic degree)
Happiness	Always, mostly, rarely, never
Pediatric Quality of Life Inventory (PedsQL)	Score
Health Utilities Index (HUI)	PROM
Malignancy	Gall bladder malignancy, gallbladder carcinoma, Ewing sarcoma, pilocytic astrocytoma, and malignant peripheral nerve sheath tumor, other (specify)
Kidney stones	Yes, no, unknown
MRI scale used in Italian studies (gene therapy trial) - Adapted Loes score	Scale from 0 - 31.5
Signs of peripheral neuropathy	Yes (>demyelinating PNP confirmed with EMG yes, no), no, unknown
Metabolic acidosis	Yes, no, unknown
Gall bladder ultrasound results	Wall thickening (>3mm), gall bladder stones, dilated bile duct (>8mm), gall bladder distension, gall bladder polyposis, collapsed gall bladder
Gall bladder resection	Yes, no, unknown
Intrathecal baclofen	Yes, no, unknown

Medication and year of start treatment	Spasmolytics, antiemetics, analgesics, anticonvulsants, antidepressants, antipsychotics, anxiolytics, sleep medication, hormone replacement therapy, immunomodulators other, none
Scale for the assessment and rating of ataxia (SARA)	Score 0 - 40
Other skeletal deformities	Scoliosis, feet deformities/contracture(s), knee contracture(s), hip contracture(s), hip luxation(s), other
Surgical intervention for skeletal/feet deformities	Yes (>specify year, indication), no, unknown
